# Salivary C-Reactive Protein in Hashimoto's Thyroiditis and Subacute Thyroiditis

**DOI:** 10.4061/2010/514659

**Published:** 2010-09-14

**Authors:** Nivedita L. Rao, Sukanya Shetty, Krishnaraj Upadhyaya, Prasad R. M, Eric C. Lobo, H. P. Kedilaya, Ganesh Prasad

**Affiliations:** ^1^Department of Biochemistry, Yenepoya Medical College, Deralakatte, Mangalore 575018, India; ^2^Department of Biochemistry, K. S. Hegde Medical Academy, Deralakatte, Mangalore 575018, India; ^3^Department of Pathology, Yenepoya Medical College, Deralakatte, Mangalore 575018, India; ^4^Department of Biochemistry, A. J. Institute of Medical Sciences, Mangalore 575004, India

## Abstract

C-reactive protein (CRP), an acute-phase reactant, has been identified as a saliva-based biomarker of inflammation. The objective of the study was to estimate and compare salivary CRP levels in Hashimoto's thyroiditis (HT) and Subacute thyroiditis (SAT). The study included 30 HT patients who presented with clinical features of hypothyroidism, 15 SAT patients who presented with clinical features of hyperthyroidism, and 20 healthy age- and sex-matched euthyroid controls. CRP levels in saliva were estimated using an Enzyme-Linked Immunosorbent Assay method with enhanced sensitivity. In HT, the mean salivary CRP levels did not differ significantly from controls. SAT patients had significantly elevated salivary CRP levels compared to HT patients and controls. The rise in salivary CRP levels in SAT patients conceivably reflects the presence of an inflammatory process. Saliva CRP levels appear to serve as inflammatory markers in SAT patients and may aid their clinical evaluation.

## 1. Introduction

Hashimoto's thyroiditis (HT) is one of the most common human autoimmune diseases responsible for considerable morbidity [1, 2]. It is characterized by diffuse lymphocytic infiltration of the thyroid gland, elevated levels of the serum antithyroid antibodies, clinical evidence of goitrous or atrophic gland, and frequent thyroid dysfunction in varying degrees [2]. Subacute thyroiditis (SAT) is an acute inflammatory disorder of the thyroid gland most likely due to viral infection [3], characterized by painful toxic goiter with systemic inflammation [4]. 

Acute-phase reactants have been implicated for their involvement as proinflammatory molecules in various inflammatory diseases [5]. Serum C-reactive protein (CRP), the classical acute-phase protein of hepatic origin, is a nonspecific marker of inflammation in the body [6, 7]. As changes in serum CRP levels reflect the presence and intensity of inflammation, CRP has been used as a clinical marker to assess the inflammatory status in many diseases. CRP estimations have not been routinely used to monitor thyroid disease, although many thyroid conditions involve inflammation. 

Saliva has been shown to contain systemically-derived biomarkers of infectious diseases [8–10]. Saliva assay systems require necessary sensitivities for the detection of biomarkers present at low, but still pathophysiologically relevant, concentrations in saliva. In the present study, a newly developed Enzyme-Linked ImmunoSorbent Assay (ELISA) method with a low limit of detection for CRP was used for its estimation in saliva. 

Previous studies have reported marked variations in serum CRP levels with different thyroid disease entities [11]. But data on CRP levels in saliva of HT and SAT patients are lacking in literature. The objective of the study was to estimate and compare salivary CRP levels in Hashimoto's thyroiditis and Subacute thyroiditis.

## 2. Materials and Methods

### 2.1. Study Groups

The patients were recruited from K. S. Hegde Charitable Hospital, Deralakatte, Mangalore, Karnataka, India.

#### 2.1.1. Hashimoto's Thyroiditis (HT) Group

It included 30 patients with the diagnosis of Hashimoto's thyroiditis. The diagnosis was based on increased antithyroglobulin antibody (Tg-Ab), antithyroid peroxidase antibody (TPO-Ab) titers, thyroid function tests, and fine-needle aspiration (FNA) cytology results.

#### 2.1.2. Subacute Granulomatous or de Quervain's Thyroiditis (SAT) Group

It consisted of 15 diagnosed patients who were negative for TPO-Ab and Tg-Ab. They presented with typical clinical symptoms such as painful tender goiter and fever. Their thyroid function test results and FNA cytology results were compatible with the diagnosis.

### 2.2. Control Group

It consisted of 20 age- and sex-matched healthy euthyroid subjects. 

Exclusion criteria for the study were the existence of any comorbid cardiac, autoimmune, infectious, musculoskeletal, or malignant disease, oral disease, and a recent history of operation or trauma. 

Women were premenopausal and none was pregnant. No individual was following any drug regimen.

### 2.3. Biochemical Evaluations

TSH and thyroid hormone levels were measured in all subjects. Serum Total T3 (TT3), Total T4 (TT4), and TSH levels were measured by Electrochemiluminescence (Elecsys 2010, Germany). Tg-Ab and TPO-Ab were measured by Solid phase enzyme immunoassay (Hycore Biomedicals Ltd, Germany). Erythrocyte sedimentation rate (ESR) was measured by Westergren method. Normal ranges in our laboratory are as follows: TT3, 0.8–1.8 ng/mL; TT4, 4.5–11.5 *μ*g/dl; TSH, 0.40–5.5 mIU/liter; Tg-Ab, less than 225 IU/mL; TPO-Ab, less than 35 U/mL; ESR, less than 20 mm/h (female), 10 mm/h (male).

### 2.4. Ethics

The procedures followed were in accordance with the ethical standards of the Institutional Human Ethics Committee and with the Helsinki Declaration. The study was approved by the Institutional Ethics Committee. Before initiation of the study, voluntary consent was obtained from each subject.

### 2.5. Collection of Saliva Samples

Unstimulated whole saliva was collected by passive drooling as described previously [12] at least 2 hours after any food intake. Briefly, after 3-4 rinses of the mouth with water, saliva was allowed to accumulate in the floor of the mouth for approximately 2 minutes and repeatedly expectorated into an ice-chilled polypropylene vial to collect about 2 mL. Following collection, the samples were stored below −20°C until their analyses.

### 2.6. Estimation of C-Reactive Protein in Saliva

The concentration of CRP in saliva was estimated by ELISA method using Salimetrics C-reactive protein ELISA kit (PA, USA).

### 2.7. Statistical Analysis

Data were analyzed with SPSS-17.00 using one-way analysis of variance (ANOVA) method and Turkey multiple comparison test. Chi-square test was used for comparison of gender differences between the study groups. *P* values lower than  .05 were considered to be statistically significant.

## 3. Results

### 3.1. Characteristics of the Groups

There was no significant difference in terms of age, gender, between the HT, SAT, and control groups (Table 1). The TSH titers of the HT patients were significantly higher when compared to SAT and control subjects. Mean TT3 and TT4 levels were below the normal lower limit in HT group indicating that the patients were in hypothyroid state. Mean TT3 and TT4 levels were above the normal upper limit in SAT group indicating that the patients were in hyperthyroid state. The ESRs with a mean value of 61.82 mm/h were significantly higher in the SAT group when compared to the control and HT groups (Table 1).

### 3.2. Cytomorphological Features Observed after FNA of Thyroid

**Figure 1:**
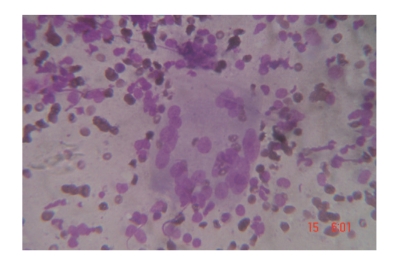
Numerous multinucleated histiocytic giant cells and scant epithelioid cells seen in thyroid tissue of woman presenting with Subacute thyroiditis (May-Grunwald-Giemsa stain; original magnification 450, reduced by 30%).

*In SAT group*, There exist epithelioid cell granulomas with multinucleate giant cells, acute and chronic inflammatory cells, follicular epithelial cells with degenerative changes, and cellular debris in the background (Figure 1). 

**Figure 2:**
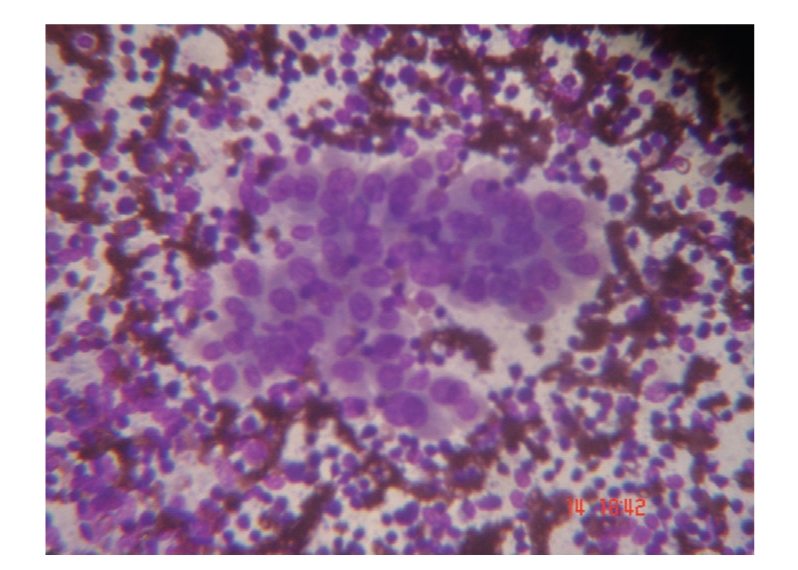
Numerous oxyphilic epithelial cells (Askanazy cells), moderate to large number of lymphocytes in a hemorrhagic background seen in thyroid tissue of woman presenting with Hashimoto's thyroiditis (May-Grunwald-Giemsa stain; original magnification 450, reduced by 30%).

*In HT group*, There exist oxyphilic epithelial cells (Askanazy cells), moderate to large number of lymphocytes, few plasma cells admixed with and surrounding the epithelial cells, variable number of epithelioid histiocytes, scanty or no colloid, and hemorrhagic background (Figure 2).

### 3.3. Saliva C-Reactive Protein Levels in HT, SAT, and Controls

Mean CRP level in saliva of HT (hypothyroid) group was not significantly different when compared to the euthyroid control group (Table 2). However, mean CRP level in saliva of SAT (hyperthyroid) group was significantly higher when compared to the euthyroid control group (Table 2). Mean salivary CRP level of SAT (hyperthyroid) group was observed to be significantly higher than the HT (hypothyroid) group (Table 2).

## 4. Discussion

The results of our study demonstrated higher salivary CRP levels in SAT patients who presented with clinical features of hyperthyroidism when compared to HT patients who presented with clinical features of hypothyroidism and euthyroid control subjects. This is not surprising because the hallmark of the laboratory evaluation of SAT is a markedly elevated ESR and elevated serum interleukin-6 (IL-6) levels [13], as well as other inflammatory markers. CRP secretion is increased in response to a complex network of cytokines, especially IL-6 and either IL-1 or tumor necrosis factor (TNF-*α*) [5]. These results of CRP in saliva are in concordance with previously reported findings by Pearce et al. that serum CRP levels were elevated in patients with painful subacute thyroiditis [11], which conceivably reflects the presence of an underlying systemic inflammatory process.

There are few data in the literature regarding the relationship between serum and saliva levels of CRP. CRP in human saliva measured by application of a microchip assay system has been shown to positively correlate with serum CRP estimated by ELISA [8]. Elevated serum and salivary CRP levels have been reported in patients with psoriasis [14, 15]. Positive correlation between porcine serum and salivary CRP has been reported [16]. In all those studies, salivary CRP concentration was able to distinguish the healthy from the diseased. The present study did not involve simultaneous serum CRP estimations. However, the above-mentioned previous reports [8, 14–16], findings of elevated serum CRP levels in SAT patients reported by Pearce et al. [11], and the demonstration of increased saliva CRP levels in SAT patients of the present study indirectly suggest the possibility of the existence of a positive correlation between saliva and serum CRP levels in the subjects. However, further studies need to be done to establish the association between serum and saliva levels of CRP.

The concentrations of most molecules present in saliva are usually one tenth to one thousandth of those in blood [9]. In the present study, the estimated mean concentration of salivary CRP in normal controls (0.837 *μ*g/L; Table 2) is observed to be approximately 1000-fold lower than the reported normal levels for serum CRP (0–8 mg/L) [17]. The estimated mean concentration of salivary CRP in the present study in HT (0.817 *μ*g/L; Table 2) is observed to be approximately 3000-fold lower than the reported mean concentration of serum CRP in HT by Pearce et al. (2.80 mg/L) [11]. According to the knowledge of the authors, there are no other reports in literature on estimation of CRP in saliva of HT or SAT patients. 

Mean CRP level in saliva of HT (hypothyroid) group of the present study was not significantly different when compared to the euthyroid control group. These results are in concordance with studies by Pearce et al. [11], Sacide et al. [18], and Kon and DeGroot [19] which reported that serum CRP levels are not significantly increased in patients with HT. However, the studies by Pearce et al. [11] and Sacide et al. [18] involved HT subjects at euthyroid status while patients of our HT group were in hypothyroid status. But the study by Kon and DeGroot [19] included patients with painful HT who presented with hypothyroidism. Therefore, CRP levels in HT appear to be independent from thyroid function status.

Regarding the association between thyroid hormone levels and serum CRP, previous studies have reflected conflicting observations. Tuzcu et al. and Christ-Crain et al. had shown a clear association between hypothyroidism and raised serum CRP [20, 21]. Taddei et al. reported higher serum CRP and IL-6 levels in patients with HT who presented with clinical features of subclinical hypothyroidism (SCH) when compared to healthy controls [22]. In contrast, Pearce et al. had shown that patients with HT, short-term hypothyroidism, and postpartum thyroiditis at different stages (thyrotoxic, euthyroid, or hypothyroid) had similar serum CRP as compared to their euthyroid controls [11]. The study by Hueston et al. also showed no difference in serum CRP levels between patients with SCH and euthyroid individuals [23]. Christ-Crain et al. reported that serum CRP levels did not correlate with thyroid hormone levels in groups of overtly and subclinically hypothyroid subjects and that treatment of subclinical hypothyroidism did not alter serum CRP values [21]. 

With respect to the matter of whether systemic inflammation exists in HT, Taddei et al. [22] hypothetically thought the inflammatory process in HT to be related to the increased TSH levels and discussed the probability of chronic activation of the immune system due to HT. Mazziotti et al. demonstrated that patients affected by HT without other apparent autoimmune disorders have a generalized activation of the immune system [24]. The study by Sacide et al. revealed low-grade systemic inflammation with extremely high values for another acute phase protein, serum amyloid A (SAA) in HT patients (euthyroid) when compared to the controls [18]. Therefore, although salivary CRP level is theoretically promising as a way to assess inflammation, it appears to have only a limited role in HT. Nevertheless, it may prove to be useful in differentiating between HT and SAT.

The acute phase reaction is in most circumstances a good indicator of inflammatory activity and tissue damage. CRP is a direct and quantitative measure for the acute phase reaction and, due to its fast kinetics, provides adequate information of the actual situation. The ESR, on the contrary, is in fact an indirect measure of the acute phase reaction. It does react much slower to changes of inflammatory activity and is influenced by a number of other factors [5]. 

The major advantages for using saliva-based assays have been described in some detail previously [8–10]. Most importantly, collection of saliva may be done by procedures that are considered to be noninvasive, painless, and convenient. Consequently, these methods may be performed several times a day. The use of saliva for CRP estimation in SAT patients could therefore allow the evaluation of their inflammatory status by using non-invasive and minimally stressful sampling methodology. 

In conclusion, CRP levels estimated in saliva of SAT patients were observed to be significantly increased compared to HT patients and euthyroid controls in our study. The rise in saliva CRP levels in SAT patients conceivably reflects the presence of an underlying systemic inflammatory process. Saliva CRP levels appear to serve as inflammatory markers in SAT patients and, therefore, may aid clinical evaluation of the patients.

## Figures and Tables

**Table 1:** Characteristics of HT, SAT, and Control Groups.

	Hashimoto thyroiditis	Subacute thyroiditis	Control group
	(*n* = 30)	(*n* = 15)	(*n* = 20)
Age (Yrs)	28.85 ± 8.83^a^	31.75 ± 10.40^a^	31.82 ± 9.39^a^
Gender (F/M)	28/2^b^	14/1^b^	17/3^b^
TSH (mIU/L)	34.25 ± 26.28^cΨ^	0.03 ± 0.02^d^	2.17 ± 1.05
TT_3_ (ng/ml)	0.48 ± 0.25^cΨ^	2.20 ± 0.36^c^	1.09 ± 0.25
TT_4_ (*μ*g/dl )	2.24 ± 1.53^cΨ^	13.43 ± 0.35^c^	8.38 ± 1.65
ESR (mm/h)	22.62 ± 8.41^dΨ^	61.82 ± 9.75^c^	18.77 ± 6.44

Data are reported as Mean ± SD

*P value <.05 is considered to be statistically significant

^a^ *P* = .456; NS (not significant)

^b^ *P* = .563; NS

^c^ *P* < .001*;* ^d^ *P* > .05; compared with control values

^Ψ^ *P* < .001; compared with SAT values.

**Table 2:** Saliva CRP Levels in HT, SAT, and Control Groups.

Group	CRP Levels *μ*g/L
Hashimoto's thyroiditis (*n* = 30)	0.817 ± 0.206^a^
Subacute thyroiditis (*n* = 15)	1.908 ± 0.752^cb^
Control (*n* = 20)	0.837 ± 0.450

Data are reported as Mean ± SD

*P* value <.05 is considered to be statistically significant

^a^ *P* = .987; NS (not significant)*;* ^c^ *P* = .001; compared with control values

^b^ *P* = .001; compared with HT values.

## References

[1] Vanderpump MP, Tunbridge WM (2002). Epidemiology and prevention of clinical and subclinical hypothyroidism. *Thyroid*.

[2] Punzi L, Betterle C (2004). Chronic autoimmune thyroiditis and rheumatic manifestations. *Joint Bone Spine*.

[3] Mendelson G (1988). Pathology of thyroid disease. *Diagnosis and Pathology of Endocrine Diseases*.

[4] Nikolai TF (1991). Silent thyroiditis and subacute thyroiditis. *Werner and Ingbar’s the Thyroid: A Fundamental and Clinical Text*.

[5] Gabay C, Kushner I (1999). Mechanisms of disease: acute-phase proteins and other systemic responses to inflammation. *New England Journal of Medicine*.

[6] Volanakis JE (2001). Human C-reactive protein: expression, structure, and function. *Molecular Immunology*.

[7] Pearson TA, Mensah GA, Alexander RW (2003). Markers of inflammation and cardiovascular disease: application to clinical and public health practice: a statement for healthcare professionals from the centers for disease control and prevention and the American Heart Association. *Circulation*.

[8] Christodoulides N, Mohanty S, Miller CS (2005). Application of microchip assay system for the measurement of C-reactive protein in human saliva. *Lab on a Chip*.

[9] Malamud D (1992). Saliva as a diagnostic fluid. *British Medical Journal*.

[10] Mandel ID (1993). A contemporary view of salivary research. *Critical Reviews in Oral Biology and Medicine*.

[11] Pearce EN, Bogazzi F, Martino E (2003). The prevalence of elevated serum C-reactive protein levels in inflammatory and noninflammatory thyroid disease. *Thyroid*.

[12] Navazesh M (1993). Methods for collecting saliva. *Annals of the New York Academy of Sciences*.

[13] Bartalena L, Brogioni S, Grasso L, Martino E (1993). Increased serum interleukin-6 concentration in patients with subacute thyroiditis: relationship with concomitant changes in serum T4-binding globulin concentration. *Journal of Endocrinological Investigation*.

[14] Chodorowska G, Wojnowska D, Juszkiewicz-Borowiec M (2004). C-reactive protein and *α*2-macroglobulin plasma activity in medium-severe and severe psoriasis. *Journal of the European Academy of Dermatology and Venereology*.

[15] Assya K, Ivan G, Aneta I (2009). Psoriatic patients and salivary components. *Oral Health and Dental Management in the Black Sea Countries*.

[16] Gutiérrez AM, Martínez-Subiela S, Eckersall PD, Cerón JJ (2009). C-reactive protein quantification in porcine saliva: a minimally invasive test for pig health monitoring. *Veterinary Journal*.

[17] Pepys MB, Hirschfield GM (2003). C-reactive protein: a critical update. *Journal of Clinical Investigation*.

[18] Sacide E, Suna B, Pervin V, Sevgin D (2008). Acute-phase reactans in Hashimoto thyroiditis. *International Immunopharmacology*.

[19] Kon YC, DeGroot LJ (2003). Painful Hashimoto's thyroiditis as an indication for thyroidectomy: clinical characteristics and outcome in seven patients. *Journal of Clinical Endocrinology and Metabolism*.

[20] Tuzcu A, Bahceci M, Gokalp D, Tuzun Y, Gunes K (2005). Subclinical hypothyroidism may be associated with elevated high-sensitive C-reactive protein (low grade inflammation) and fasting hyperinsulinemia. *Endocrine Journal*.

[21] Christ-Crain M, Meier C, Guglielmetti M (2003). Elevated C-reactive protein and homocysteine values: cardiovascular risk factors in hypothyroidism? A cross-sectional and a double-blind, placebo-controlled trial. *Atherosclerosis*.

[22] Taddei S, Caraccio N, Virdis A (2006). Low-grade systemic inflammation causes endothelial dysfunction in patients with Hashimoto's thyroiditis. *Journal of Clinical Endocrinology and Metabolism*.

[23] Hueston WJ, King DE, Geesey ME (2005). Serum biomarkers for cardiovascular inflammation in subclinical hypothyroidism. *Clinical Endocrinology*.

[24] Mazziotti G, Sorvillo F, Naclerio C (2003). Type-1 response in peripheral CD4+ and CD8+ T cells from patients with Hashimoto's thyroiditis. *European Journal of Endocrinology*.

